# Draft Genome Sequence of *Nitrobacter vulgaris* Strain Ab_1_, a Nitrite-Oxidizing Bacterium

**DOI:** 10.1128/genomeA.00290-17

**Published:** 2017-05-04

**Authors:** Brett L. Mellbye, Edward W. Davis, Eva Spieck, Jeff H. Chang, Peter J. Bottomley, Luis A. Sayavedra-Soto

**Affiliations:** a Department of Botany and Plant Pathology, Oregon State University, Corvallis, Oregon, USA; b Molecular and Cellular Biology Program, Oregon State University, Corvallis, Oregon, USA; c Biocenter Klein Flottbek, Department of Microbiology & Biotechnology, University of Hamburg, Hamburg, Germany; d Department of Microbiology, Oregon State University, Corvallis, Oregon, USA

## Abstract

Here, we present the 3.9-Mb draft genome sequence of *Nitrobacter vulgaris* strain Ab_1_, which was isolated from a sewage system in Hamburg, Germany. The analysis of its genome sequence will contribute to our knowledge of nitrite-oxidizing bacteria and acyl-homoserine lactone quorum sensing in nitrifying bacteria.

## GENOME ANNOUNCEMENT

Aerobic nitrification is generally a two-step process where ammonia is oxidized to nitrite, which is subsequently oxidized to nitrate (1). The second step is carried out by nitrite-oxidizing bacteria (NOB) (2, 3). NOB include both *r*-strategists, such as *Nitrobacter* spp., and *K*-strategists, such as *Nitrospira* spp., which coexist in a variety of environments (2–4). *Nitrobacter* spp. play a role in the response to large nitrogen fluctuations in soils and other systems (5–7). In addition, *Nitrobacter* spp. were the first NOB shown to produce and respond to acyl-homoserine lactone (AHL) quorum-sensing (QS) chemical signals (8, 9). *Nitrobacter vulgaris* strain Ab_1_ is a well-studied nitrifier, yet it has no available genome sequence (5, 10, 11). To address this need, we sequenced the genome of *Nitrobacter vulgaris* strain Ab_1_. Our primary goal was to identify loci corresponding to AHL autoinducer synthase and AHL-binding LuxR transcription factors.

Genomic DNA was isolated using the Wizard genomic DNA purification kit (Promega). A Nextera XT DNA sample preparation kit was used to construct the sequencing library. The instructions were followed, up to those for normalization of libraries. A Qubit double-stranded DNA high-sensitivity assay kit (Life Technologies, Inc.) and Agilent TapeStation 4200 high-sensitivity D5000 DNA ScreenTape (Agilent Technologies) were used to determine the concentration and average sizes of the library fragments. The library was then quantified by quantitative PCR on an ABI 7500 Fast real-time system (Life Technologies, Inc.) using the Kapa library quantification kit (Kapa Biosystems). Sequencing was completed on a MiSeq (Illumina) 250-bp paired-end nano flow cell.

There was a total of 2,436,208 reads, for an average coverage of 156×. Nextera XT adapter sequences were trimmed from the raw reads using the BBDuk software, as recommended in the manual (http://jgi.doe.gov/data-and-tools/bbtools/). Reads were error-corrected and assembled into contigs using SPAdes version 3.10.0, with the “--careful” flag and the k-mer setting of “-k 21,33,55,77,99” (12), and screened for contaminating sequences with the blobtools software (version 0.9.19.5) (13, 14). *De novo* assembly of the MiSeq reads resulted in 95 contigs that totaled 3,900,573 nucleotides in length, with a mean contig size of 41,059 nucleotides; the *N*_50_ contig length was 130,999 nucleotides. Genome annotation was completed using the NCBI Prokaryotic Genome Annotation Pipeline, resulting in 3,501 coding genes and 56 RNA-coding genes (15). The *N. vulgaris* genome sequence is 59.8% G+C and has pairwise average nucleotide identities (16) of 83.0% and 81.2% to *Nitrobacter winogradskyi* and *Nitrobacter hamburgensis*, respectively (17, 18). These low values suggest that *N. vulgaris* is too distant from comparators to be considered a member of their species.

The *N. vulgaris* genome has all the genes necessary for chemolithotrophic growth on nitrite. Interestingly, genes encoding a putative AHL autoinducer synthase and AHL-binding LuxR homolog were present, as well as putative nitric-oxide-forming *nirK* (*aniA*) and *nnrS* genes, possibly suggesting similar QS regulation of NO fluxes to *N. winogradskyi* (9).

### Accession number(s).

The genome of *N. vulgaris* strain AB_1_ was deposited at DDBJ/EMBL/GenBank under the accession number MWPQ00000000. The version described in this paper is the first version.
